# Breast implant-associated squamous cell carcinoma in a male patient: A case report and review of the medical literature

**DOI:** 10.3389/fsurg.2022.983611

**Published:** 2023-01-10

**Authors:** Zihuan Xia, Bing Han, Lei Wang, Guansen Ning, Zongke Guo, Jue Zhang, Bing Yu, Ming Chen, Wanxing Zhang, Ke Wang, Xiaojun Ma

**Affiliations:** ^1^ Department of Plastic Surgery, Nanjing Tongren Hospital, Southeast University, Nanjing, China; ^2^ Department of Plastic Surgery, Zhongda Hospital, Southeast University, Nanjing, China

**Keywords:** squamous cell carcinoma, breast implant, male breast cancer, Poland's syndrome, case report

## Abstract

**Background:**

Primary squamous cell carcinoma (SCC) of the breast is a rare tumor type. The diagnosis of this tumor type is more frequently made only after microscopy evaluation. Breast implant-associated SCC is rarer with medical literature review indicating only 18 cases reported in female individuals.

**Case presentation:**

We reported an unusual case that a man found a 3-cm sized mass on his left breast at first, who had a implant surgery 18 years previously to reconstruct the deformed left breast, as related to the Poland's syndrome. More than 1 year after the mass was detected, the size of the mass gradually increased to 20 cm with swelling and severe pain, and the patient was admitted to our hospital. The patient underwent surgical excision of the tumor, followed by removal of the implant, complete capsulectomy, and sentinel lymph node biopsy. The microscopy evaluation demonstrated the tumor as moderately and poorly differentiated invasive SCC. Follow-up at 12 months after showed multiple metastases, including the skin of the chest, axillary lymph nodes and pleura.

**Conclusion:**

Breast implant-associated SCC can occur in male patients. Therefore, it should be considered when the clinical manifestation or histopathological appearance is not typical of other breast neoplasms. Malignant transformation of normal epithelial cells takes about 18 years, after which rapid evolution follows leading to fast growth of the tumor.

## Introduction

Primary squamous cell carcinoma (SCC) of the breast is a rare and generally aggressive disease. Primary SCC represents less than 0.1% of invasive breast carcinomas, characterized by large tumor size, rapid progression, frequent relapse, and defavorable prognosis. SCC associated with breast implant surgery has been rarely reported. The medical literature shows less than 20 cases of SCC arising from capsular tissue around the breast implant (1–10). All cases reported are of female patients. No male patient cases have been reported yet. Here, we first reported an unusual male case of breast implant-associated SCC.

## Case presentation

The patient was a 52-year-old male, who initially found a 3-cm sized mass in the left breast region. He had a history of Poland's syndrome and underwent unilateral breast prosthesis implantation 18 years previously to reconstruct the deformed left breast. More than 1 year after the mass was detected, the patient started to have continued swelling and increased pain as the multinodular mass gradually increased to the size of 20 cm. Serial positron emission tomography–computed tomography (PET–CT) revealed undetermined primary mass on the capsule surrounding the breast implant. No other lesions were observed in skin, oral cavity, respiratory tract, gastrointestinal tract, bladder, and other areas.

**Figure 1:**
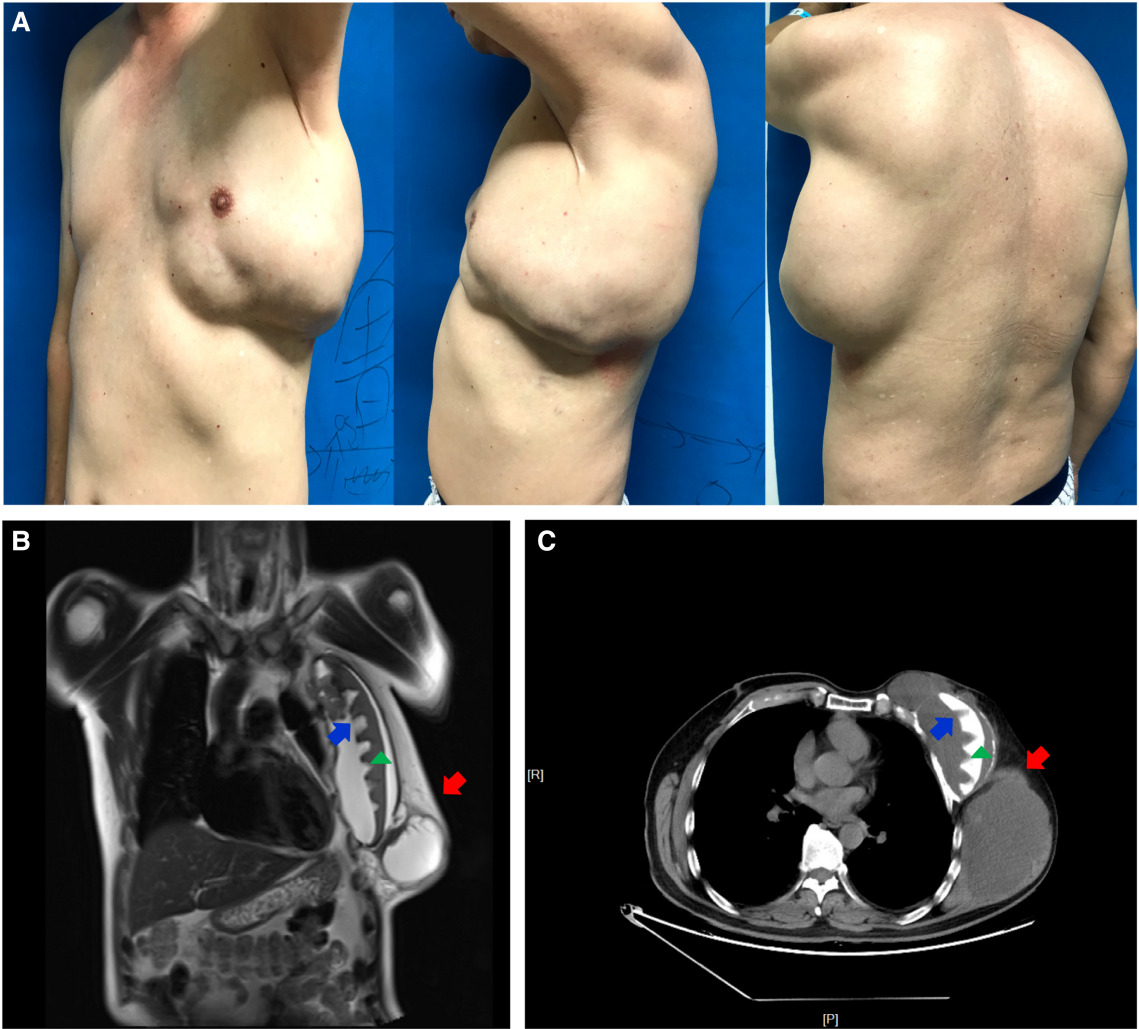
Physical examination and imaging studies of the patient. A large-sized multinodular mass was on the left chest of a 52-year-old man with a breast implant for past 19 years (**A**). MRI (**B**) and CT (**C**) findings showed a 20-cm sized polycystic mass (red arrow) in front of the implant (green triangle) and multiple irregular masses (blue arrow) in front of the ribs posterior to the implant.

Physical examination at the time of admission revealed a 25 cm × 12 cm × 8 cm multinodular mass on the left breast and chest wall with an irregular surface uplift. The skin aspect was normal, without inflammation, ulceration, pigmentation, etc. (Figure 1A). No record of breast tumor was found among the patient's family members. Routine clinical biochemistry and laboratory tests failed to show any abnormal index.

Magnetic resonance imaging (MRI) performed at the time of admission showed that the pectoralis muscle in the left chest was congenitally absent. A 20-cm sized polycystic mass was seen in front of the breast implant. Moreover, multiple irregular masses were observed between implant and ribs. Localized bone destruction occurred in the 3rd, 4th and 5th ribs ribs, accompanied by a large amount of wrapped liquid around the prosthesis and the outer and posterior chest. The bilateral axillary lymph nodes were enlarged with bilateral pleural thickening and a small amount of hydrothorax in the left pleural cavity. Furthermore, CT demonstrated a similar result (Figures 1B,C).

Sonography revealed a heterogeneous multinodular mass. Multiple papillary projections were observed posterior to the implant. The nature of these nodules was undetermined. However, an ultrasound-guided biopsy revealed that no malignant tumor cells were found in the cystic fluid and section of cell block.

**Figure 2:**
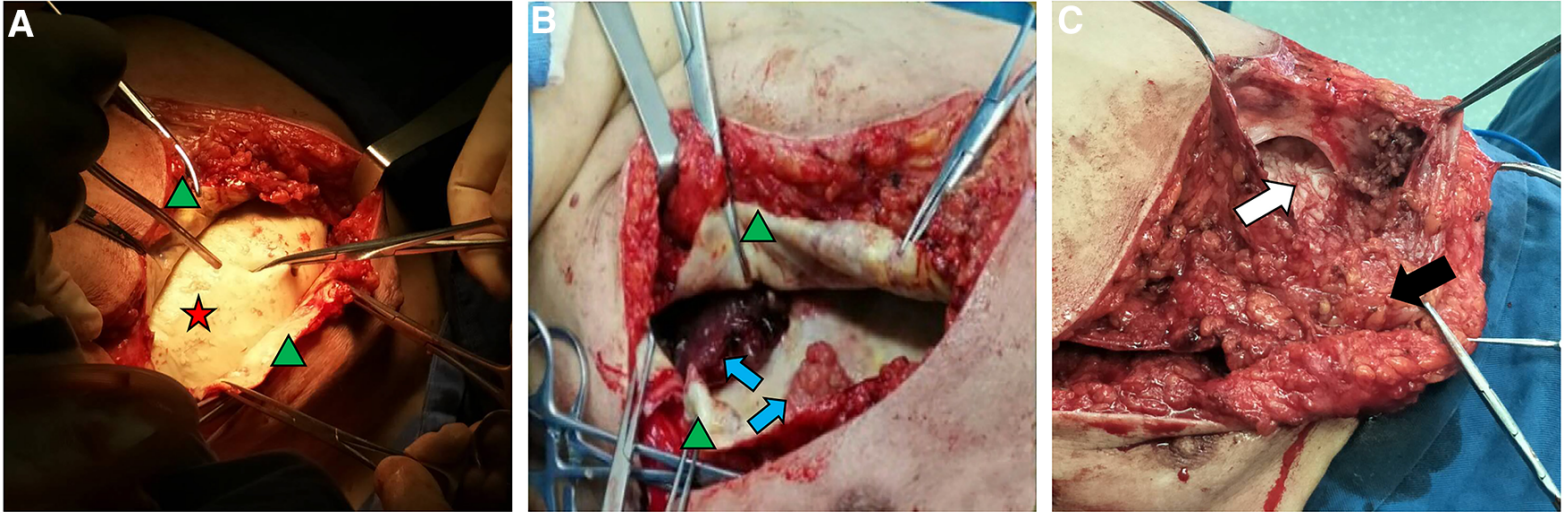
Intraoperative appearance of the implant and the mass. The implant (red star) was intact (**A**); Formation of capsule (green triangle) and presence of several heterogeneous nodules composed of liquid-fillen spaces in which solid papillae project (blue arrow) were observed (**B**); A polycystic tumor was in front of the implant composed of solid tumor zones (black arrow) and cystic tumor zones (white arrow) (**C**).

**Figure 3:**
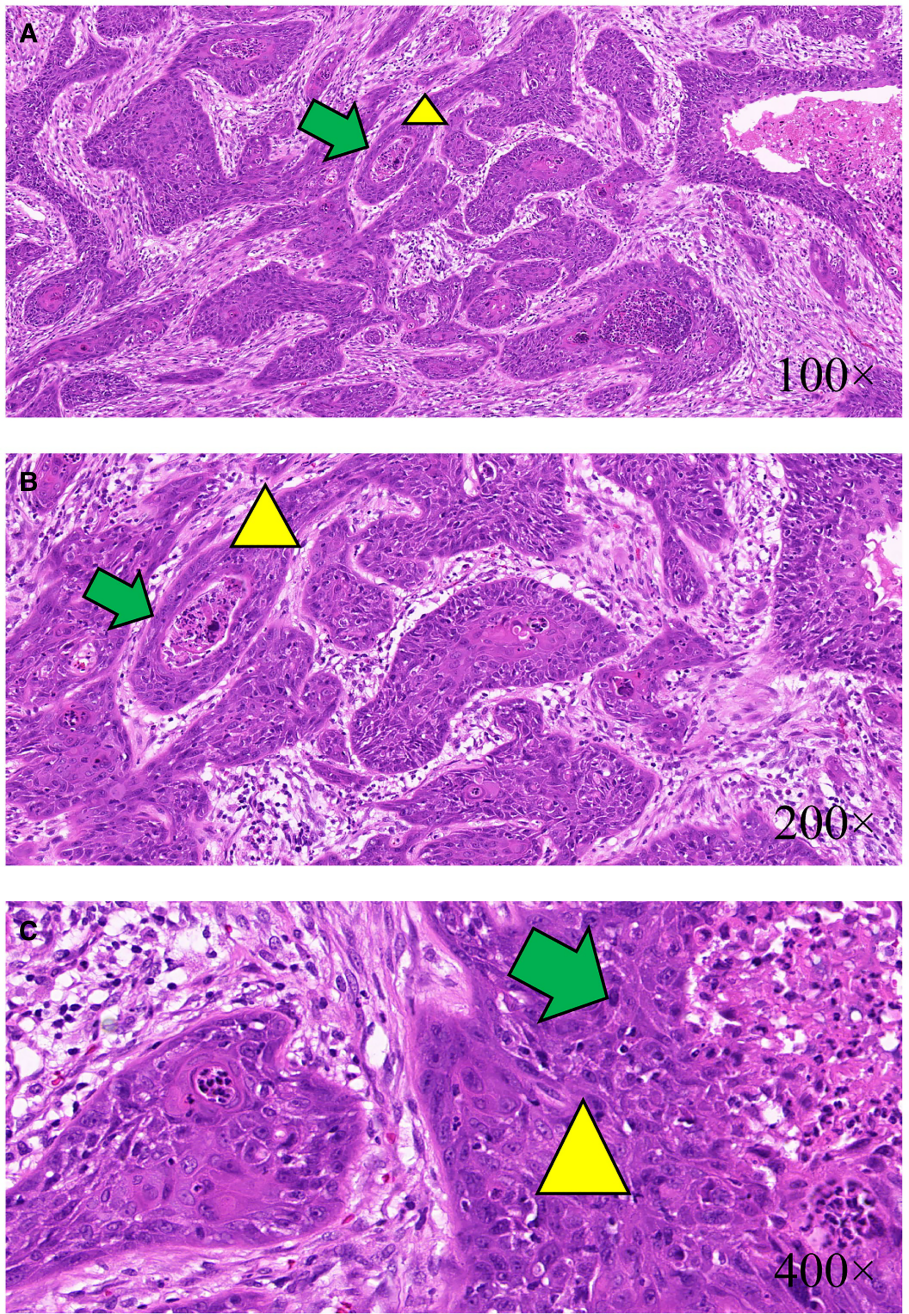
Histology studies of the papillary projections and the mass. The papillary projections and the mass were demonstrated as moderately and poorly differentiated SCC (yellow triangle) arising from the implant capsule. Squamous cells aggregated to form cancer nests (green arrow); H&E ×100 (**A**), H&E ×200 (**B**), and H&E ×400 (**C**).

**Figure 4:**
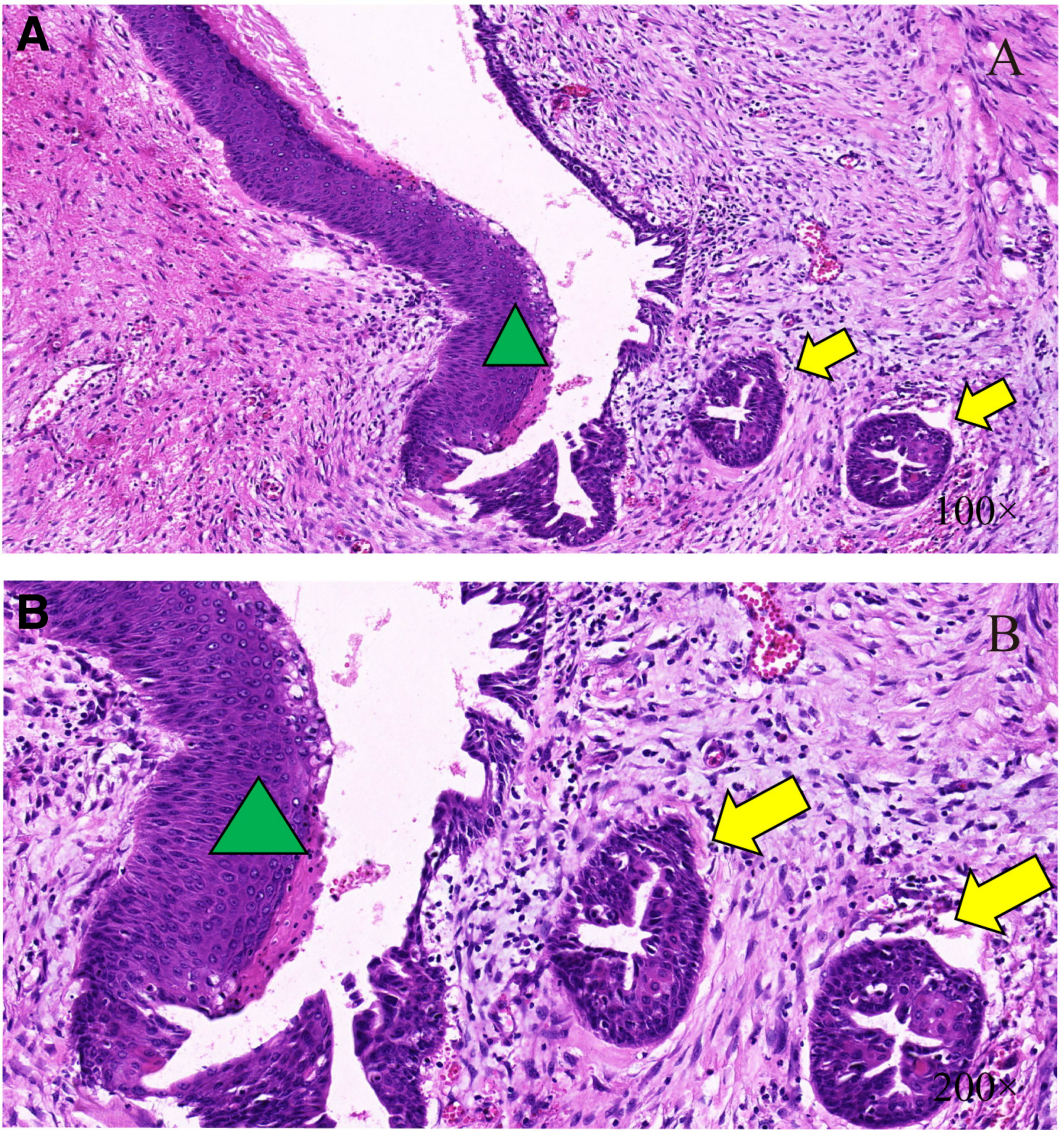
Histology studies of the duct epithelium. The male mammary duct epithelium (green triangle) was local squamous metaplasia, forming cysts (yellow arrow); H&E ×100 (**A**) and H&E ×200 (**B**).

After completing all examinations, the patient received tumor excision surgery, implant removal surgery, complete capsulectomy, and sentinel lymph node biopsy. Fibrous tissue envelope was observed on the surface of the implant during the operation. The implant was of textured surface type with a volume of 150 cc, and the name of the manufacturer was unknown. The tumor was a heterogeneous multinodular mass in front of the implant, with a volume of about 1,500 cc. The mass was polycystic, with irregular structure and a large amount of clear liquid in the fibrous tissue capsule. After excision of the capsule and the huge polycystic mass, the surgeon removed the implant. Multiple irregular masses were detected in front of the ribs posterior to the implant. It was observed that papillary projections invaded into the thoracic cavity and left axilla (Figure 2). Therefore, the nodules and the sentinel lymph node were resected and sent for pathological examination together with the voluminous tumor and the capsule. The final microscopy assessment revealed that the papillary projections were moderately and poorly differentiated SCC arising from the implant capsule and measuring 2.5–3.5 cm (Figure 3). Squamous metaplasia of duct epithelia was observed in the capsule, as well as acute and chronic inflammation, and fibrous tissue hyperplasia (Figure 4). The tumor was negative for estrogen receptor, progesterone receptor, HER2/neu, and calponin, and positive for Ki67, P16, P40, and P63 (Supplementary Figure S1). The sentinel lymph node tested negative for metastatic disease. After the surgery, the patient was transferred to the Oncology Department for further adjuvant systemic therapy and radiotherapy. At the request of the patient and his family, the patient was discharged on September 14, 2020 and accepted adjuvant systemic therapy at a local hospital. The systemic therapy was unknown. Follow-up within a year indicated a defavorable prognosis and occurrence of multiple metastases, including the skin of the chest, axillary lymph nodes and pleura.

## Discussion

According to the World Health Organization Classification of Breast Tumors, diagnosis of primary SCC requires that: (1) more than 90% of the malignant cells show squamous differentiation, (2) there are no other primary sites of SCC, and (3) the lesion must be separated from the skin and nipple (11). Primary SCC of the breast is a rare diagnosis, accounting for less than 0.1% of all breast cancers. Breast implant-associated primary malignancies are also extremely rare, with less than 20 cases reported (1, 2, 3, 6, 9, 10, 12).

In the reported cases of implant-associated SCC and the current study, the patient had no clinical evidence of primary SCC of the breast or cutaneous or distant invasive SCC, as such the reported cases have met these criteria. All the patients in the previously reported case studies were females; however, in the current study, the patient was male. Hence, it has been proved, for the first time, that not only females but also males have the risk of developing implant-associated SCC after undergoing breast implantation surgery.

The histogenesis of implant-associated SCC of the breast is still unclear. Hypotheses included metaplasia from benign disease of the breast parenchyma, malignant growth of previously quiescent intrinsic epidermal elements (such as an epidermal cyst), and chronic abscess (6). Multiple case studies have suggested that silicone implant placement can lead to metaplastic squamous epithelium, which may be a precursor to SCC. Malignant squamous transformation is known to develop in wounds involved with chronic inflammation. However, there is no experimental or clinical evidence to support expected findings of epithelialization of the implant capsule and subsequent squamous dysplasia in the pathologic evaluation of capsulectomy specimens (13–15). In this case study, we present a rare case of SCC of the breast with a rare mechanism of onset. Our patient had no evidence of primary mammary carcinoma or SCC from other sites. Pathology report of the implant capsule revealed that multiple “epidermoid cysts” were formed, a large number of acute and chronic inflammatory cells were infiltrated, fibrous tissue was proliferated significantly, and squamous epithelial metaplasia and carcinogenesis of male mammary duct tissue had occurred in some areas. In one place, squamous metaplasia that continued with duct epithelium and formation of cystic lesions with dysplastic squamous cells were observed. Different from poorly differentiated invasive squamous cells in other parts, cells in this invasive zone are moderately differentiated. This was observed in the capsule tissue of the prosthesis, rather than residual breast tissue. The tumor didn't show vascular emboli. We speculated that mammary duct epithelia migrated around the implant or transferred to the prosthetic capsule during surgery or capsule formation, and subsequently, underwent squamous metaplasia and carcinogenesis gradually under the long-term stimulation of chronic inflammation.

In previously reported cases as well as in the current study, the patient had a remote history of breast implant surgery (>15 years ago) with silicone prosthesis (1–3). The result suggested that it takes at least 15 years for squamous cell metaplasia and carcinogenesis to occur in epithelium. In a previously reported case, based on the findings the researchers suggested the occurrence of a chronic inflammatory process, through which epithelial cells may turn dysplastic over a period of 10–20 years (1–3). In the current study, the patient found a tumor 18 years after implantation. And the tumor grew rapidly within 1 year, increasing in size from 3 to 20 cm. These data indicate that malignant transformation of normal epithelial cells takes about 18 years, and the rapid proliferation of cells after malignant transformation led to rapid growth of the tumor. Compared with the long process before, the rapid evolution suggested that it was a tumor with high malignancy. One year after tumor resection, multiple metastasis of tumor were confirmed by telephone follow-up. The rapid course and multiple metastasis highlight both the aggressive nature of disease and its defavorable prognosis. Given the aggressiveness of the disease, early diagnosis may be the key to improving disease-specific survival. Primary SCC arising from a breast implant capsule is exceedingly rare and lacks typical clinical manifestations in the early stage, which makes it easy to be missed and misdiagnosed. The reported case showed that the breast implant-associated SCC might occur in male patients as well similar to female patient. Therefore, diagnosis of SCC should be considered when the clinical manifestation or histopathological appearance is not typical of other breast neoplasms. As with most of the cases of SCC, the tumors were negative for breast markers, suggesting that these tumors are resistant to hormone treatments. The patient in this study is male, suggesting that the formation of tumor is not affected by estrogen and progesterone. In previously reported cases as well as the current study, most of the patients underwent negative sentinel lymph node biopsies. The presence of widespread multiple metastases post-surgery may suggest a more aggressive hematogenous metastasis mechanism and an inadequacy of local control, which underscores the importance of incorporating adjuvant systemic therapy.

## Conclusion

Primary SCC arising from a breast implant capsule is an exceedingly rare occurrence without previously reported male case. The reported case shows that breast implant-associated SCC may occur in not only female patients but also male patients. There should be a high index of suspicion for SCC of the breast in patients with a history of breast implants. It needs at least 15 years for the normal cells to become cancerous, and rapid evolution follows after malignant transformation, which leads to fast growth of the tumor. Although the etiology of breast implant-associated SCC remains unclear, a possible mechanism could be the chronic inflammation that leads epithelia in the capsular tissue or the compressed residual breast tissue to squamous cell metaplasia and dysplasia. If the diagnosis of SCC is confirmed, aggressive systemic therapy as well as surgery may be needed to improve future outcomes.

## Data Availability

The original contributions presented in the study are included in the article/**Supplementary Material**, further inquiries can be directed to the corresponding authors.
