# Supplementary figures and images for: Breast implant-associated squamous cell carcinoma in a male patient: A case report and review of the medical literature
Source: Front Surg. 2023 Jan 10;9:983611. doi: 10.3389/fsurg.2022.983611 (PMC9871623; doi:10.3389/fsurg.2022.983611)

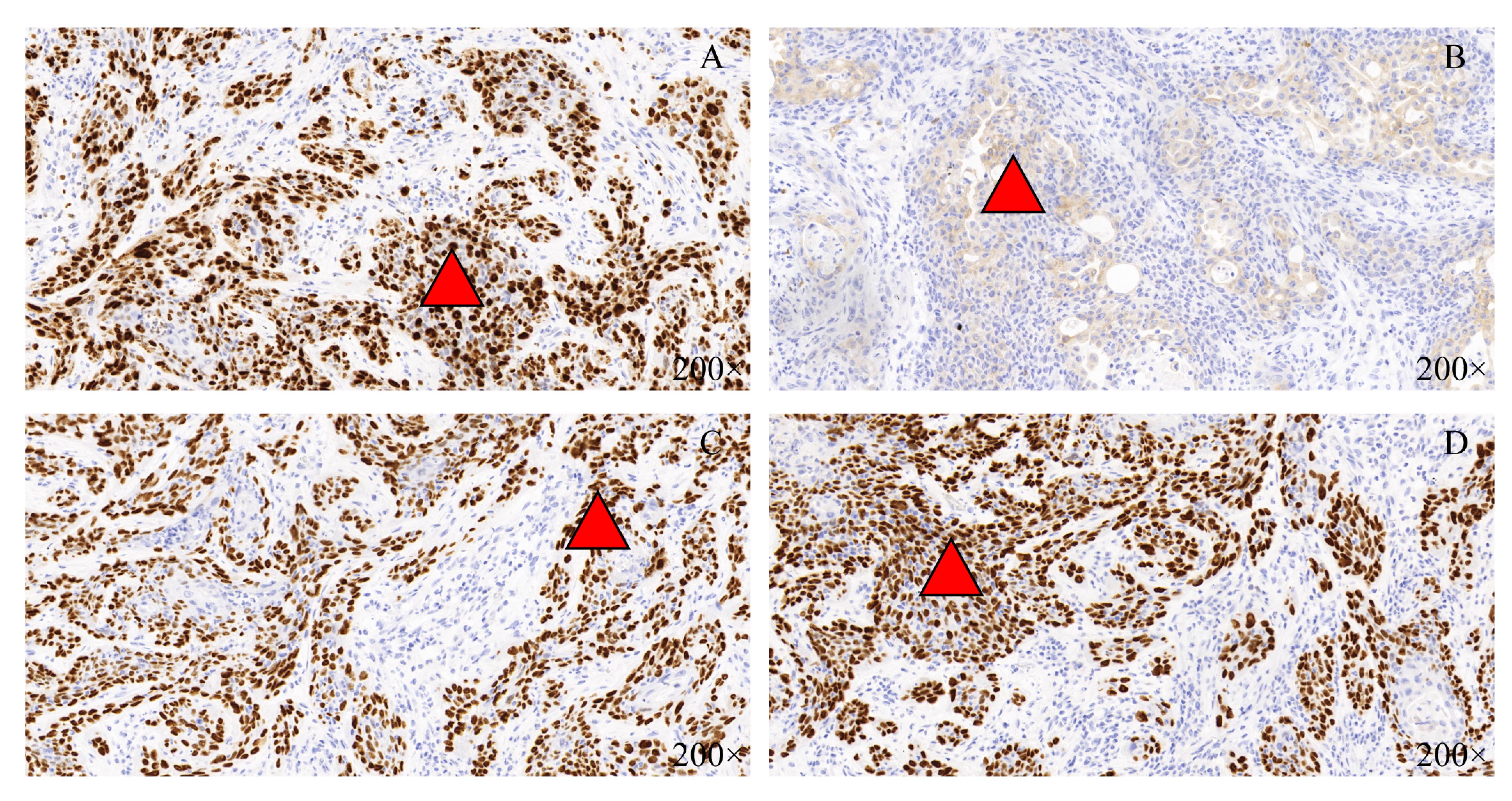

Supplement: Supplementary file 1 [file Image1.jpeg]
